# Antimalarial medicine preference and usage in rural and peri-urban communities in Lagos and Osun states in southwestern Nigeria

**DOI:** 10.5281/zenodo.10732924

**Published:** 2017-02-22

**Authors:** Monday Tola, Ojo Oreoluwa, Emmanuel T. Idowu, Esther O. Iyede, Olusesan Omidiji, Taiwo S. Awolola

**Affiliations:** 1 Public Health Division, Nigerian Institute of Medical Research, Lagos, Nigeria; 2 Department of Zoology, University of Lagos, Lagos, Nigeria; 3 Department of Cell Biology and Genetics, University of Lagos, Lagos, Nigeria; 4 Laboratory Unit, Badagry General Hospital, Badagry, Lagos, Nigeria

## Abstract

**Background:**

Medicine preference, usage and health-seeking behaviour are very important in the treatment of malaria and prevention and management of drug resistance.

**Materials and methods:**

A descriptive cross-sectional study, using a semi-structured questionnaire administered to 135 respondents, was carried out to assess antimalarial drug preference and usage among rural dwellers in Alajue, Ede, Osun State and peri-urban dwellers in Ajara, Badagry, Lagos State, Nigeria.

**Results:**

Loss of appetite, fever, chill and rigour, headache and vomiting were the most frequently reported symptoms (83.3%, 78.6%, 71.4%, 69.0% and 64.3%, respectively). More than half (57.1%) of the respondents had their drugs prescribed by a qualified health practitioner. Sixty-eight (50.3%) respondents treated malaria with Artemisinin-based Combination Therapy (ACT) while Sulphadoxine-Pyrimethamine (SP), paracetamol and herbal medicine usage was reported by 11.9%, 9.6% and 4.4% of the respondents, respectively. Thirty-two respondents (23.7%) took nothing to treat the infection. Of them, only 64.3% completed their drugs regimen during their last episode with 35.7% reporting that fever subsided on/before day 2 of treatment and 64.3% reported that fever subsided two days post treatment. The majority (83.3%) of respondents had no adverse reaction to the drugs used (16.7% reported drowsiness, nausea, headaches and vomiting) with 64% of the respondents reporting that they will use ACT again anytime they have malaria and about 65% reported that the drug was very convenient for them (χ^2^ = 18.192, *p* = 0.001).

**Conclusions:**

The control of drug resistance in malaria parasites requires reducing the overall drug pressure, improving the ways the drugs are used and prescribing follow-up practices. The use of drug combinations that are not likely to foster resistance like ACT is also a good measure of resistance control. ACT would be expected to remain the key anti-malarial drug for treatment of multidrug resistance *P. falciparum* since there are no alternative drugs available at present.

## 1 Introduction

Malaria remains a major global public health concern with one third of the global population at risk of infection. According to the latest estimates by the World Health Organization (WHO), 198 million cases of malaria occurred globally in 2013 and the disease led to 584 000 deaths [1]. The burden is heaviest in Africa where an estimated 90% of all malaria deaths are reported African children below 5 years of age account for 78% of all deaths [1]. Malaria is also of global concern because of its high economic burden on endemic countries, high prevalence of mortality in children and pregnant women, as well as non-immune individuals [2].

Malaria impedes human development and thus having social consequences and a heavy burden on economic development with Nigeria, losing over ₦132 billion ($694.7 million) from cost of treatment and absenteeism from work, school and farm [3]. Studies in Africa have shown that the initial treatment of malaria fever often takes place at home without consulting trained health professionals. Inappropriate behaviour can interfere with the effectiveness of a control measure, such as vector control or chemotherapy [3]. These issues are particularly important in tropical areas where malaria control options are limited because of the parasite and vector resistance to antimalarial drugs and insecticides, respectively. In such cases, an understanding of the communities' beliefs and behaviour may be crucial to the success of specific control measures [4]. Informal use of antimalarial drugs could increase the risk of under-dosage, over-dosage or incorrect dosing, treatment failure, resistance to antimalarial drugs, occurrence of adverse drug reactions and drug interactions which could compromise effective antimalarial treatment [5].

Lack of knowledge about rational use of antimalarial drugs among patients is a serious problem, especially in areas of intense transmission, where antimalarial drugs are given repeatedly to treat fevers (even in the absence of malaria), thus increasing the risk of resistance and adverse drug reactions [6]. Thus, the main aim of treatment for malaria is to reduce morbidity and mortality and also delay the development of antimalarial drug resistance. The attitude of the community and correct use of these drugs are very important to treatment and prevention of emergence of parasites resistant to treatment. Considering the increased prevalence of parasites that are resistant to many antimalarials, there is a need to determine antimalarial drug usage and preference in the selected regions and study population [5].

## 2 Materials and methods

A descriptive cross-sectional survey was carried out to assess antimalarial drug preference among people in the selected communities of Ajara, Badagry (Lagos State) and Alajue, Ede (Osun State), southwestern Nigeria. Badagry (6025’N 2053’E) is a coastal town in Lagos State with an area of 440 km2 and a population of 241,093. It was founded on a lagoon off the Gulf of Guinea with its protected harbour leading to the town making it a key port during the slave trade era. Badagry is also a border town with the Republic of Benin at Seme. Ede (7040’N 4030’E), is an ancient Yoruba town, located in the guinea savannah zone. It has a total area of 336 km2 and a population of 159,866. The predominant occupation of the people of Ede (especially Alajue village) is farming and about 90% of the people are Muslims.

A semi-structured questionnaire, specifically developed for the purpose of gathering information on drug preference, was used for data collection. The sample size was determined by using the formula n = Zα2pq/d2 [7] and the total sample size was 135 after adding a 20% contingency. Data were collected in August 2014. Parents or caregivers of children under 5 years of age were interviewed on behalf of their wards. Inclusion criteria were those that gave their consent to be part of the study, had malaria or were diagnosed with malaria and had used (or were currently using) antimalarial drugs for treatment, were aged 2 years or above and were recruited at a health facility.

Ethical approval was obtained from the Nigerian Institute of Medical Research Review Board (1RB/13/218). Informed consent form was also given before the respondent was given time to fill the questionnaire and non-educated were assisted with filling the questionnaire. Data were analysed using SPSS version 20. Descriptive statistics was done using tables showing frequencies and percentage distributions. The variables that were significantly associated with pattern/preference (dosage completion and drug preferences) were further analysed using Chi-square tests. A p-value of < 0.05 was considered statistically significant.

## 3 Results

### 3.1 Socio-demographic characteristics

Of the 135 respondents interviewed, 42 (31.0%) were in age group of 6-10 years and, most (97, 71.8%) were students. A large proportion 100 (73.8%) was single. The respondents’ socio-demographic characteristics are shown in Table 1.

**Table 1.** Socio-demographic characteristics of respondents (n=135).

Characteristic	N	%
**Age Group**		
≤5	32	23.8
6-10	42	31.0
11-20	16	11.9
21-30	19	14.3
≥31	26	19.0
**Occupation**		
Student	97	71.8
Trading	32	23.7
Farming	6	4.4
**Gender**		
Male	74	54.8
Female	61	45.2
**Marital Status**		
Single	100	73.8
Married	35	26.2

### 3.2 Respondents’ awareness of signs/ symptoms of uncomplicated malaria

Chills and rigour (96; 71.4%), vomiting (87; 64.3%), headache (93; 69%) and loss of appetite (113; 83.3%) were the most mentioned signs/symptoms of malaria among the respondents or parents/caregivers while paleness of the eyes (3; 2.4%) and body weakness (3; 2.4%) were mentioned the least.

### 3.3 Drug use and effect

Half of the respondents reported that they experienced fever two weeks preceding the study. Of these, half (50%) reported that they took ACT while 23.8% reported no drug use. Dosage completion was high (64.3%, Table 2).

**Table 2.** Type of drugs used for malaria treatment by respondents (n=135).

Variable	N	%
**Medication taken**		
ACT	68	50.4
SP	16	11.9
Paracetamol	13	9.6
Herbal medicine	6	4.4
Nothing	32	23.7
**Place of drug acquisition**		
PMV	109	80.7
Roadside	26	19.3
**Dosage completion**		
Yes	87	64.3
**Non-completion (reasons)**		
Fever subsided	13	27.1
Drug not convenient	9	18.7
No improvement	10	20.8
Reaction to drug	3	6.3
No reason	13	27.1

PMV = Patent Medicine Vendors

### 3.4 Adverse reaction to drugs and drug preferences

Eighty-three percent of the respondents claimed they did not react to the drug while 60% expressed their willingness to take the drug again due to its effectiveness and being recommended by a doctor (Table 3).

**Table 3.** Occurrence and type of adverse reactions to medication taken.

Variable	N	%
**Episode of adverse reaction(s)**		
Yes	23	16.7
**Types of reaction**		
Drowsiness	9	39.1
Nausea	5	21.7
Headaches	6	26.1
Vomiting	3	13.1
**Would you use drugs again for malaria treatment?**		
Yes	80	59.5
**If yes, why would you treat malaria with the same drug?**		
Doctor’s recommendation	3	2.4
It is effective	77	57.1
**If no, why would you not treat malaria with the same drug?**		
Has side effects	3	2.4
No improvement	13	9.5
No response	39	28.6

### 3.5 Dosage completion

Drug convenience, source of drug prescription, occupation, if fever subsided, all had statistical significance with willingness to complete drug dosage (Table 4).

**Table 4.** Dosage completion amongst the respondents (n=135).

Variable	Was the dosage completed?				
	Yes (%)	No (%)	χ^2^	df	p-value*
**Occupation**			8.28	2	0.041
Student	71 (73.3)	26 (26.7)			
Trading	10 (31.3)	22 (68.7)			
Farming	6 (100)	0 (0.0)			
**Source of drug prescription**			24.87	3	<0.001
Doctor’s prescription	74 (95.8)	3 (4.2)			
Self-prescription	10 (23.1)	32 (76.9)			
Friend	0 (0.0)	6 (100)			
Others	3 (33.3)	7 (66.7)			
**Was the drug convenient?**			13.14	1	<0.001
Yes	67 (87.5)	10 (12.5)			
No	19 (33.3)	39 (66.7)			
**Did fever subside?**			11.54	1	<0.001
Yes	83 (76.5)	26 (23.5)			
No	3 (12.5)	24 (87.5)			

*Significant difference (P<0.05)

### 3.6 Drug preference

Medication taken, source of drug prescription, drug convenience, days before fever subsided and reaction to drug all had statistical significance with the choice of drug taken (Table 5).

**Table 5.** Drug preference among the respondents (n=135).

Characteristic	Would you like to take the drug again?				
	Yes (%)	No (%)	χ^2^	df	p-value*
**Medication taken**			22.24	1	<0.001
ACT	64 (80.0)	16 (20.0)			
Others	3 (5.9)	52 (94.1)			
**Source of Drug Prescription**			21.31	3	<0.001
Doctor	65 (84.0)	12 (16.0)			
Self	15 (35.3)	27(64.7)			
Friend	0 (0.0)	6 (11.8)			
Others	6 (8.0)	4(5.9)			
**Drug Convenience**			18.19	1	<0.001
Yes	65 (84.0)	12 (17.6)			
No	10 (16.0)	48 (82.4)			
**Reaction to drug**			7.14	1	0.008
Yes	27 (4.0)	8 (35.3)			
No	108 (96.0)	15 (64.7)			

*Significant difference (P<0.05)

## 4 Discussion

Chloroquine and SP resistance in *P. falciparum* has been reported widely in Nigeria and led to a change in the National guidelines for the treatment of malaria from chloro-quine/SP to ACT as the first-line drug in 2004 [8]. Increasing evidence suggests a decline in the efficacy and some degree of resistance of *P. falciparum* to artemisinins in the Greater Mekong sub region. Early evidence came from western Cambodia and the Thai-Cambodian border in patients following treatment with either artesunate monother-apy or artesunate-mefloquine [9-12].

Drug use pattern can serve as a means to identify causes/origins of drug resistance. The knowledge about the signs/symptoms of malaria among the respondents in our study revealed that most of the reported symptoms included loss of appetite, followed by fever, headache and vomiting, which are the classical symptoms of malaria. Health education, targeted at mothers/caregivers who reported symptoms such as stomach ache, skin rash, paleness of the eyes and weakness in children as malaria signs should be conducted. These findings are consistent with studies from Ghana [13] and Tanzania [14] in which knowledge on symptoms of malaria was similar. The study in Tanzania investigated the prevalence of malaria among individuals seeking treatment for fever and/or malaria at drug stores, showing that 24% of the people who experienced fever and reported to drug stores to purchase antimalarial drugs were actually diagnosed with malaria [14].

The findings from this study revealed that the national guideline for malaria treatment is being followed. However, a few respondents still use Sulphadoxine/ Pyrimethamine (SP) as the first line drug for treatment of malaria. There is therefore a need to educate these communities to always visit a hospital if they observe signs and symptoms of malaria and adhere to the prescription given as 57% of clinicians prescribe according to the national guideline. This was contrary to earlier reports from Nigeria which stated that only 5.9% of antimalarial prescriptions from hospitals contained ACTs despite the high proportion of doctors that claimed to follow national antimalarial policy change [15], especially in private hospitals [16].

Fifty-five percent of the respondents that had malaria were below 10 years of age. This shows that children are most vulnerable to malaria. A study conducted in Lagos State, Nigeria reported that about 70% of parents and infants tend to sleep under insecticide-treated bednets (ITNs) [17]. Several national surveys have shown persistently low levels of ownership and use of ITNs. The proportion of Nigerian households that owned at least one ITN was 2.2% in 2003 and 8% in 2008, while the proportion of under-five children who used ITN during the same periods was 1.2% and 5.5%, respectively [18]. Arogundade *et al.* (2011), from their study in Nigeria, reported other factors significantly associated with the use of bednets such as education, geopolitical zone and misconception about causes and prevention of malaria [19]. Alaii *et al.* (2003), in her study from Kenya, reported that the use of bednets was seasonal and was only meant for adults, not children [20]. Proper mosquito screens need to be employed in most homes and health education on malaria prevention methods should be given at schools.

Our survey showed that 57% of the respondents had a doctor’s prescription for the treatment of malaria. This act should be encouraged as doctors in hospitals tend to only prescribe ACT and development of delay in parasite clearance or resistance can be easily detected and reported. Self -medication was carried out by 30%, with 7% buying antimalarial drugs by following television and radio commercials while 5% purchased drug based on a friend’s prescription. There is need for increase in health education to discourage people from the act of self-medication because this could lead to the development of drug resistance [21].

Our findings were similar to the findings of Jombo *et al*., which showed that self-medication through procurement of antimalarial drugs over the counter without prescription was recorded for 34% of the respondents [22] but contrary to a study which reported that the commonest groups of medication prone to self–medication include antimalarials and antibiotics [23]. The study revealed that the most common drug used by the respondents for the treatment of malaria was ACT. About 50% treated malaria with ACT, 11.9% with SP and 23.8% claimed they did nothing about the infection. The use of orthodox antima-laria drugs over the traditional remedies is very high in this study. This could be attributed to the fact that the majority of respondent consulted a health worker.

The majority of the respondents purchased their drugs from patent medicine vendors such as hospitals, pharmacies or chemist stores while others got their drugs from a roadside dealer. Roadside drug dealers are a major source of counterfeit and sub-therapeutic drug dispensers. Place of purchase could also interfere with behavioural pattern of respondents to antimalarial drug use. Drugs purchased from hawkers or market places could be detrimental to health. These habits could lead to drug resistance in *P. falciparum* and illegal trading of antimalarial drugs should therefore be discouraged by the regulatory authorities. A good number of the respondents (64.3%) completed the drug regimen while those that did not gave reasons such as subsided fever, no improvement or the drug not being convenient. This shows that there is more need for drug adherence counselling or education by health practitioners to their patients, disappearance of signs/symptoms does not mean cure. This finding is contrary to the study of Omole and Onademuren in Abeokuta [24], who reported that Sul-phadoxine/Pyrimethamine (SP) combinations such as Amalar™ and Fansidar™ are frequently purchased antimalarial drugs followed by chloroquine. Artesunate mono-therapy is the most frequently purchased of the artemisinin derivatives [24]. In spite of resistance report of artesunate monotherapy in the six geo-political zones in Nigeria [25], it is disappointing that many respondents still prefer using the drug for malaria treatment. The level of ACT use in this study is far lower than that reported in a similar study in Tanzania (Dar-es-salaam City), where 87% of the respondents’ prescriptions contained Artemether-Lumefantrine [26].

Pattern of prescription in the study shows a clear preference for ACT as drug of choice which indicates a high conformity to policy recommendation. Drug use pattern can serve as a means to identify causes of resistance. The survey also revealed that due to the efficacy and convenience of ACT, 80% of the study population indicated that they will take the drug again whenever they have malaria. The reason given by the respondents treating malaria with ACT next episode was that it was very effective. Preference for ACT is consistent with many study findings in both Nigeria and other African countries [27-29]. However, majority of respondents (83.3%) did not report any adverse drug reaction. In another study, general body weakness, blurred vision or dizziness experienced by some respondents that took artesunate plus amodiaquine, could also have been due to the amodiaquine component since some of the documented side effects of 4-aminoquinoline antimalarial agents includes dizziness, general weakness, blurred vision and Fatigue [30].

Analysis of the relationship between drug used and parameters like age and occupation was significant indicating that the age bracket, 11-20, mostly used ACT while 21-30 mostly used other drugs. The data also showed that doctors would rather prescribe ACT and prescription of respondents friends were other types of drug used in the treatment of malaria. Sixty-one percent (61%) of those that used ACT claimed that fever subsided and this was within the first 2 days. The results also showed that 81.1% would like to take ACT again while 77.8% will not use the other type of antimalarial they have being using again. Contrary to this study is a survey done in Oyo State, Nigeria which stated that only 16.7% of respondents would want to take ACT again, not minding the high cost, as long as they find the drug effective [31].

## 5 Conclusions

The predominant antimalarial medicine used in the study communities is ACT, especially amongst those that visited health facilities. This is likely due to the effectiveness of ACT and government policy to provide the drug at an affordable price. ACT can be expected to remain the key anti-malarial drug for treatment of multidrug resistance *P. falciparum* since there is no alternative drug available at present. There is therefore a need for behaviour change communication in the communities on the proper use of antimalarial. Continuous monitoring and surveillance of clinical efficacy of ACT, including identification of true artemisinin-resistant parasites, is required for appropriate implementation of malaria control policy in Nigeria.
